# City Initiative: Baby Steps to a Better Future

**DOI:** 10.1371/journal.pmed.0030505

**Published:** 2006-11-28

**Authors:** Ramesh Vidavalur

**Affiliations:** University of Connecticut Health Center, Farmington, Connecticut, United States of America

The article on the new initiative for newborn health in India by A. Fernandez and D. Osrin [1] outlines an innovative approach to tackling the alarming global burden of infant mortality. There are currently an estimated 8 million annual deaths within the first month of life [2], the majority of which are in developing countries. Dr. Fernandez rightly focuses on challenges and the possible innovative, yet effective, interventions to improve maternal and infant health.

Historically, it almost took 50 years to reduce the infant mortality rate in India by 50% (151 per 1,000 live births in 1951 to 69 per 1,000 live births in 2001), and the policy makers are optimistic about further decreasing it to 30 per 1,000 live births by 2010. They also hope to reduce the present maternal mortality rate of 4/1,000 to 1/1,000 by the same time [3]. As the majority of reproductive and childhood morbidities and mortalities can be reduced by primary prevention and early intervention strategies, large-scale adaptation of community intervention strategies will be particularly useful in helping the next generation to have a head start in good health, as the expected population in 15–59 age group will massively increase from 519 million to 800 million by 2016 in India [4].

Health spending in India at 6% of gross domestic product is among the highest levels estimated for developing countries. In per capita terms, it is higher than in China, Indonesia, and most African countries, but lower than Thailand. Public spending on health in India has itself declined from 1.3% of gross domestic product in 1990 to 0.9% in 1999. Individual states have cut down health budgets from 7.0% to 5.5%, in spite of the Bhore Committee (a committee on health surveys and development) recommendation of 15% [5]. The lack of resources, infrastructure, and awareness can further hinder the balance of health and disease and puts a considerable burden on the poor, rural Indian population.

In recent years, a growing constituency of nongovernmental organizations have drawn attention to maternal and child health, supplementing government's relatively recent initiative Child Survival and Safe Motherhood. As Fernandez et al. rightly point out regarding the demand and supply sides, India is not likely to be in a position to afford institutional care for all births even if this was considered a desirable goal. So the supply side should be strengthened through birth attendants, community education, microlevel organizations, and the effective integration of traditional and modern health-care systems. One of the best ways to do this is through identifying and strengthening nongovernmental organizations that have high motivation, social commitment, sensitivity to poor, flexibility, and innovativeness. They can mobilize, empower, and increase awareness through their grassroot workers from the communities.

It has been proven in numerous trials that community-based interventions such as home-based neonatal care and usage of birth attendants can significantly reduce the infant mortality rate and improve maternal health [6].

At present, India is in the midst of an epidemiological and health transition wherein multiple factors like urbanization and migration, changing lifestyles, and democratic decentralization have an enormous impact on the above issues. Now is the time for governments and policy makers not only to put feasible plans in place with short-term goals, but also to ensure that we have adequate human resources, strong community ownership, improved information dissemination through the media, investment in health systems research, and lastly, a system that will monitor progress towards a healthy future.
